# *De novo* transcriptome analysis and gene expression profiling of an oleaginous microalga *Scenedesmus acutus* TISTR8540 during nitrogen deprivation-induced lipid accumulation

**DOI:** 10.1038/s41598-018-22080-8

**Published:** 2018-02-27

**Authors:** Anchalee Sirikhachornkit, Anongpat Suttangkakul, Supachai Vuttipongchaikij, Piyada Juntawong

**Affiliations:** 1 Special Research Unit in Microalgal Molecular Genetics and Functional genomics, Department of Genetics, Faculty of Science, Kasetsart University, Bangkok, Thailand; 2 Center for Advanced Studies in Tropical Natural Resources, National Research University-Kasetsart University, Bangkok, Thailand

## Abstract

Nitrogen deprivation (−N) has been used as a technique to promote lipid accumulation in various microalgae. *Scenedesmus acutus* is a promising oleaginous green microalga that can be cultivated in organic wastewater for biodiesel production. Nevertheless, the molecular mechanisms controlling *S*. *acutus* lipid accumulation in response to −N remain unidentified. Physiological study determined that −N reduced cell growth and photosynthetic pigments. On the other hand, it promoted carbohydrate and neutral lipid accumulation. To find the mechanisms underlying lipid accumulation, we performed *de novo* transcriptome profiling of the non-model *S*. *acutus* in response to −N. The transcriptome analysis revealed that glycolysis and starch degradation were up-regulated; on the contrary, gluconeogenesis, photosynthesis, triacylglycerol (TAG) degradation and starch synthesis were down-regulated by −N. Under −N, the carbon flux was shifted toward fatty acid and TAG synthesis, and the down regulation of TAG lipase genes may contribute to TAG accumulation. A comparative analysis of the −N transcriptomes of oleaginous microalgae identified that the down-regulation of multiple lipase genes was a specific mechanism found only in the −N transcriptome of *S*. *acutus*. Our study unraveled the mechanisms controlling −N-induced lipid accumulation in *S*. *acutus*, and provided new perspectives for the genetic manipulation of biodiesel-producing microalgae.

## Introduction

Higher economic growth is positively associated with an increase in petroleum consumption. Microalgae are well-known as a potential source for biodiesel production. They are efficient oxygenic photosynthetic organisms that are easy to cultivate and are fast growing. Despite several advantages, microalgae-based biodiesels are still far from being an economically-sustainable replacement for fossil fuels due to their low yield and high cost of production^1–3^.

Nitrogen deprivation (−N) has been commonly used as an efficient method to induce lipid accumulation in microalgae^4–8^. In the unicellular green alga model, *Chlamydomonas reinhardtii*, −N stimulates gametogenesis and zygosporulation at the expense of growth arrest^9^, while lipid bodies and starch granules are being accumulated as food storage that provides energy during spore germination. Indisputably, algal biodiesel production would benefit from the uncoupling of lipid accumulation from growth impediment.

*Scenedesmus acutus* is a fresh-water oleaginous microalga that has a high potential for biodiesel application due to its ability to grow in organic wastewater and to accumulate high lipid content^10,11^. Damiani *et al*.^12^ demonstrated that *S*. *acutus*, when cultivated autotrophically, increases cellular lipid storage, mainly triacylglycerol (TAG) following −N. The fatty acid composition of the *S*. *acutus* TAG is suitable for biodiesel feedstock with a high percentage of oleic acid (46.97%)^12^. To date, the molecular genetic basis orchestrating lipid accumulation in *S*. *acutus*, especially under −N, remains unknown.

In recent years, studies in model oleaginous microalgae, including *C*. *reinhardtii* and *Nannochloropsis oceanica*, revealed that −N-induced lipid accumulation can cause extreme alterations at the transcriptomic, proteomic, and metabolomic levels^5,6,8,13–16^. It has been proposed that the intrinsic ability to produced large quantities of lipid is species-specific^3^. However, the complete identification of the mechanism controlling lipid biosynthesis in different oleaginous species that reflects the genetic distinctions affecting the production of lipid, is still lacking. Transcriptomic analysis of the non-model microalgae typically requires short RNA sequence (RNA-seq) reads. In most studies, the RNA-seq experiments were designed for *de novo* transcriptome assembly for gene discovery and metabolic pathway reconstitution purposes. To describe how cells respond to a particular treatment, a genome-wide gene expression profiling approach must be applied to obtain a global picture of the cellular responses to a certain condition. Therefore, *de novo* transcriptome analysis, in combination with gene expression profiling, could provide a basic understanding of the molecular responses to −N-induced lipid accumulation in a non-model *S*. *acutus*.

This study aims to characterize the molecular responses to −N-induced lipid accumulation from the non-model microalga *S*. *acutus*. To this end, we applied *de novo* transcriptome and differential gene expression analyses to the RNA-seq data. Finally, we comparatively examined −N-induced transcriptomes from other oleaginous green microalgae in order to identify conserved- and organism-specific responses that result in the enhancement of lipid accumulation. Lastly, we discuss the genetic modification approaches to target some candidate genes for increasing TAG accumulation in *S*. *acutus*.

## Results and Discussion

### Physiological and metabolic adjustments of *S*. *acutus* in response to −N

**Figure 1:**
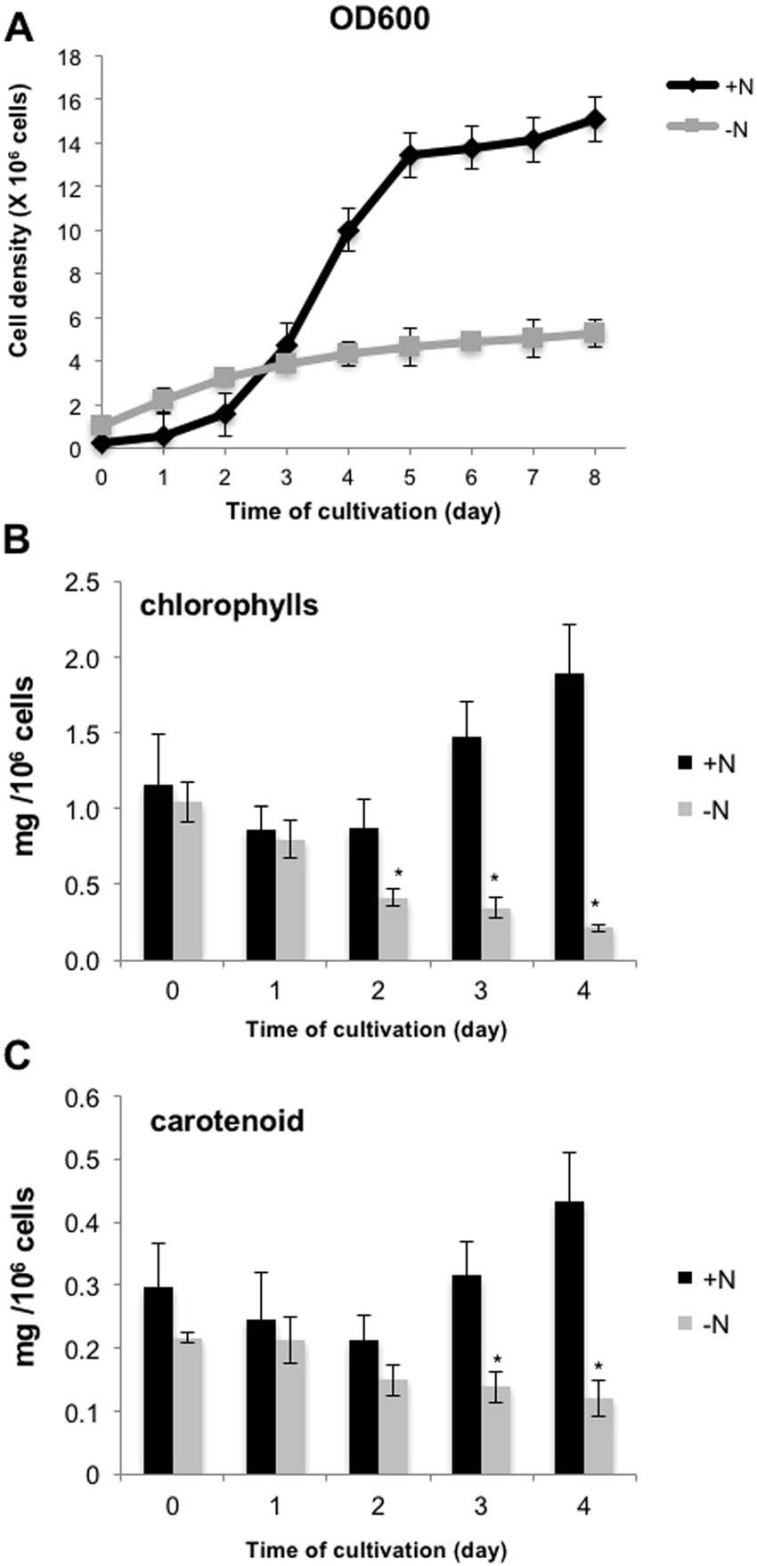
Physiological responses of *S*. *acutus* to nitrogen deprivation. *S*. *acutus* cultures were grown in TAP (+N) and TAP-N (−N) medium. (**A**) Cell density was determined by measuring the optical density at 600 nm. (**B**) Chlorophyll contents. (**C**) Carotenoid contents. Results are shown as means ± SD (*n* = 3). *p < 0.05, **p < 0.01 (*t*-test).

To examine the response of *S*. *acutus* to nitrogen availability, *S*. *acutus* was mixotrophically grown in TAP, with and without a nitrogen supply, for N-repletion (+N) and N-depletion (−N), respectively. Under +N, the cell density increased exponentially from day 1 (0.58 × 10^6^ cell/mL) to day 5 (13.43 × 10^6^ cell/mL). It plateaued after day 5, indicating that cell division occurred early during the 5-day period. On the other hand, the −N cell density was highest at day 8 (5.28 × 10^6^ cell/mL; Fig. 1A), suggesting that −N impacted cell growth. In addition, the reduction of the total chlorophyll and carotenoid contents was observed (Fig. 1B and C). There was a slight fall in total chlorophyll and carotenoid during days 1 to 2 in both +N and −N conditions. However, the +N culture gradually increased its chlorophyll and carotenoid contents after day 3, while the −N culture failed (Fig. 1B and C). The reduction of cell growth and photosynthetic pigments is regularly observed in several microalgal strains cultivated under −N^5,6,17–21^.

**Figure 2:**
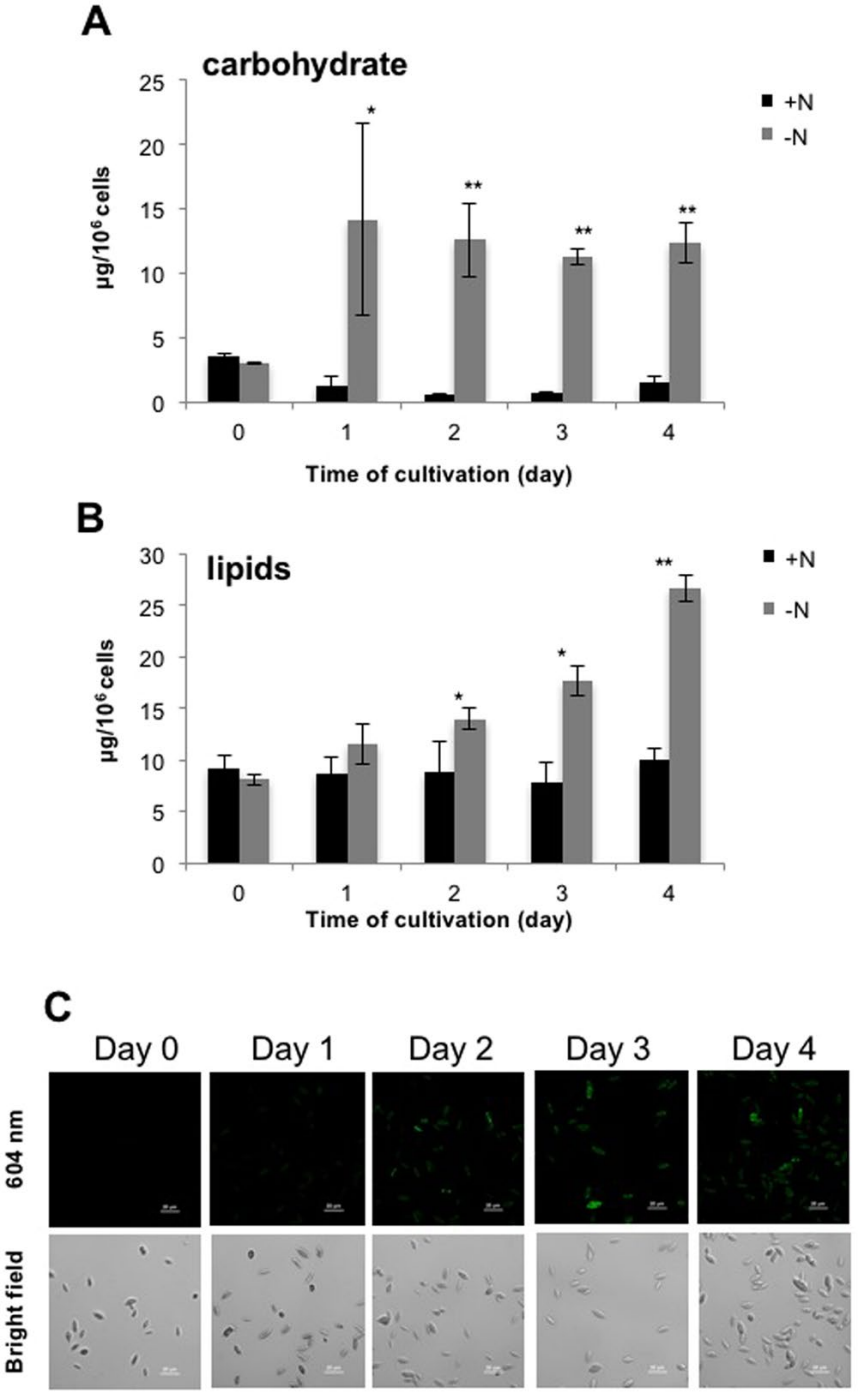
Characterization of carbohydrate and lipid contents. *S*. *acutus* cultures were grown in TAP (+N) and TAP-N (−N) medium. (**A**) Carbohydrate contents. (**B**) Lipid contents. (**C**) Neutral lipid in −N culture monitored by nile red staining at 604 nm. Results are shown as means ± SD (*n* = 3). Scale bars represent 20 µm. *p < 0.05, **p < 0.01 (*t*-test).

Next, we examined the effect of −N on total carbohydrate and lipid accumulation. In our study, total carbohydrate increased and maintained since day 1 (Fig. 2A). In contrast, the total lipid increased slowly starting from day 2 in the case of −N (Fig. 2B). The carbohydrate and lipid content of the +N culture remained constant during the entire experiment (Fig. 2A and B). Remarkably, under −N, carbohydrate accumulated faster than did lipid (Fig. 2A and B). Moreover, intracellular neutral lipid detection by nile red staining, showed that the neutral lipid accumulation increased sharply on day 2 of −N cultivation (Fig. 2C). Our results are correlated with the previous study of −N cultivation in *S*. *acutus*^12^. It has been proposed that under −N, photosynthesis induces stress by overflow of photosynthetic energy, causing oxidative stress damage and promoting carbohydrate and lipid accumulation as energy sinks^3,22^.

### *De novo* transcriptome assembly and annotation

**Figure 3:**
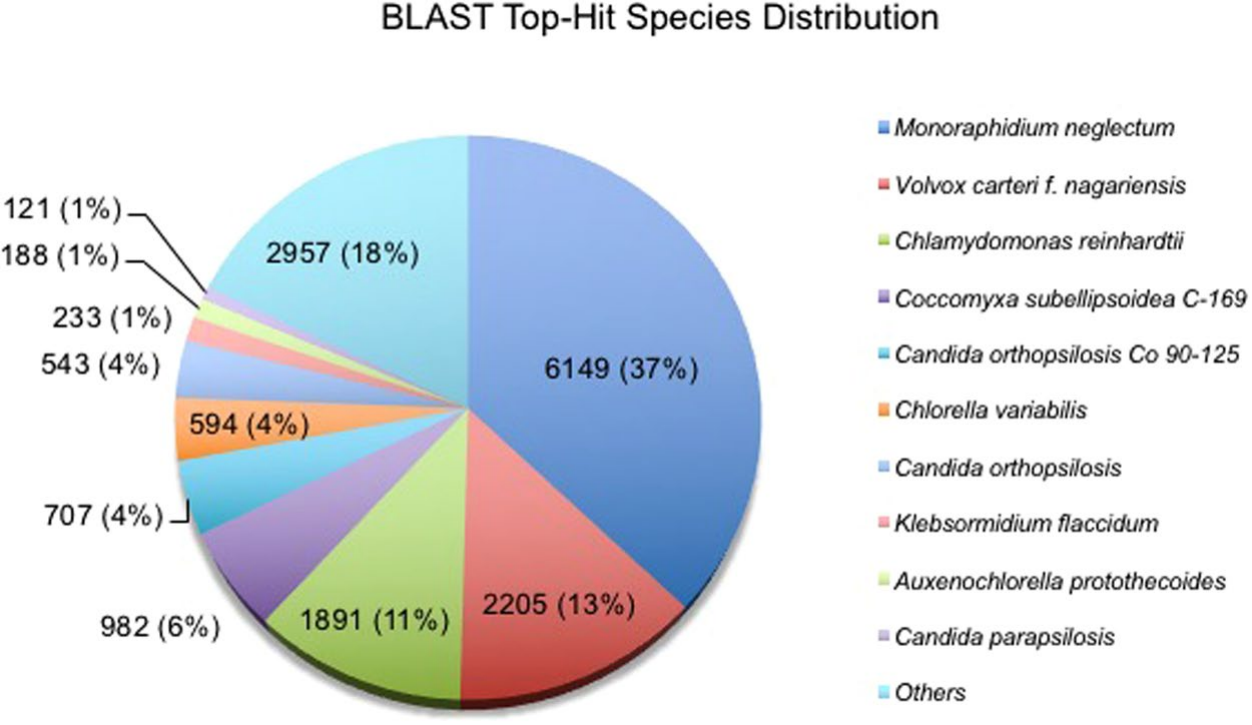
Distribution of BLAST top hit species. Percent distribution of the 10 top-hit species identified by BLAST against NCBI non redundant protein database.

To capture the transcriptome changes during the lipid accumulation phase, RNA-seq analysis was performed using +N and −N samples derived from day 2 cultures. The RNA-seq reads from four libraries (two biological replicates per each condition) were combined and subjected to *de novo* transcriptome assembly by Trinity. The transcriptome assembly yielded 51,846 transcripts with the N50 of 1,351 bp and an average transcript length of 824 bp (Supplementary Table 1). Subsequently, the assembled transcripts were annotated by BLASTX against a non-redundant (NR) protein database. Of the 51,846 transcripts, 15,461 transcripts had at least one significant hit, identified by BLASTX searching. More than 75% of the significant hits came from green algae such as *Monoraphidium neglectum* (37%), *Volvox carteri* (13%) and *Chlamydomonas reinhardtii* (11%) (Fig. 3).

To classify the function of the assembled transcripts, Gene Ontology (GO) assignment was carried out. In the “Biological Process” category, the top three GO terms were “metabolic process”, “cellular process”, and “single-organism process” (Supplementary Figure 1A). In the “Molecular Function” category, the top three GO terms were “cell”, “cell part”, and “organelle” (Supplementary Figure 1B). In the “Cellular Component” category, the top three GO terms were “catalytic activity”, “binding”, and “transporter activity” (Supplementary Figure 1C). To further analyze the transcript functions, KEGG pathway mapping was undertaken. Over 500 enzyme codes were assigned to the assembled transcripts (Supplementary Table 1). All the required enzymatic genes in *de novo* fatty acid and TAG (triacylglycerol) biosynthesis were identified from the *de novo* transcriptome data (Supplementary Figure 2; Supplementary Table 1).

### Overview of the transcriptome adjustment of *S*. *acutus* in response to −N

**Figure 4:**
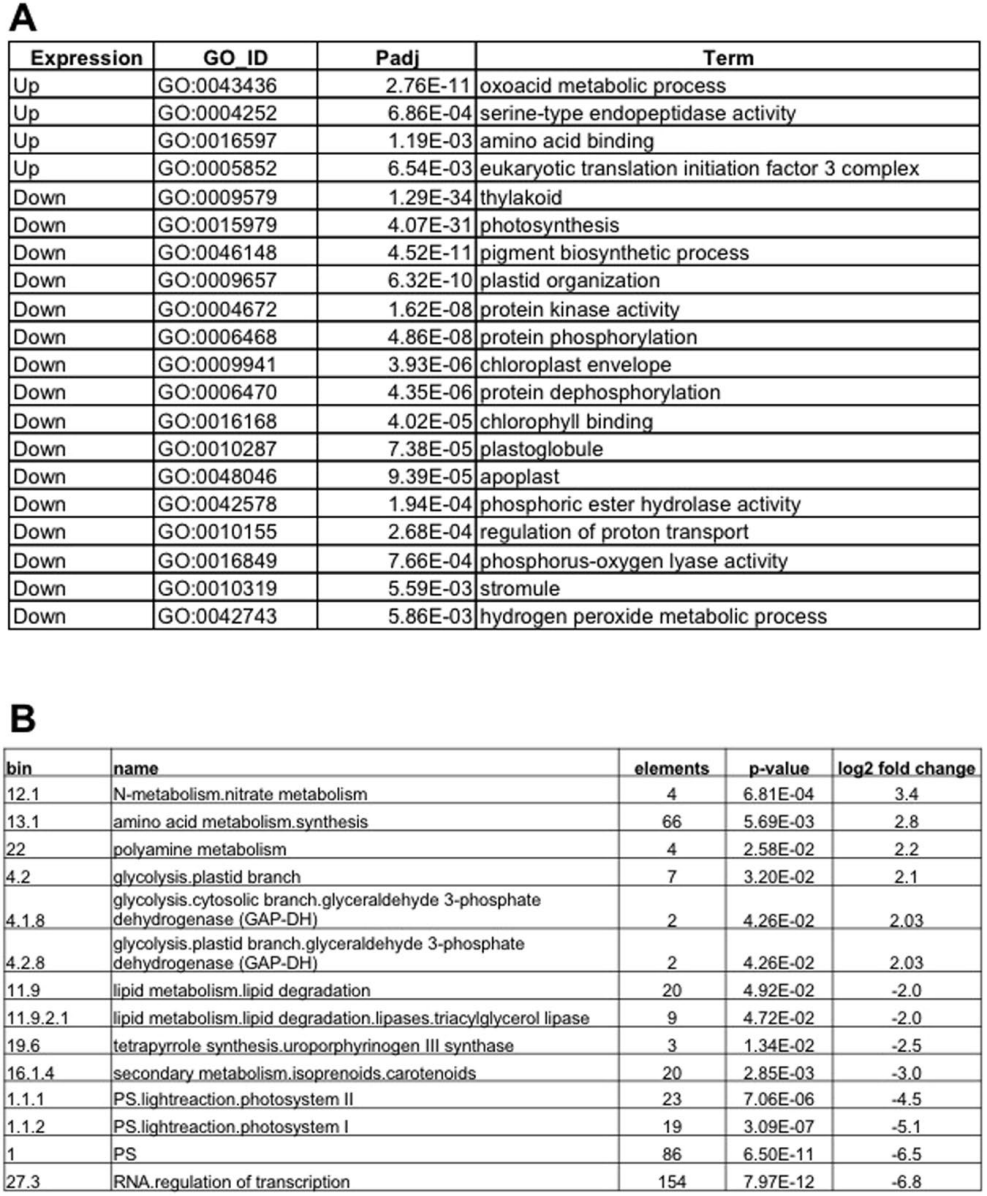
Differential gene expression during the −N-induced lipid accumulation phase in *S*. *acutus*. (**A**) Gene ontology enrichment results from up- and down-regulated transcripts are shown using an adjusted *p*-value (Padj) cuttoff < 0.001. (**B**) MAPMAN enrichment results with significant up- or down-regulation bins (*p-*value < 0.05). Data used to generate this figure can be found in Supplementary Table 2.

The two independent biological replicates of RNA-seq reads from the +N and −N samples were mapped to a *de novo* assembled transcriptome using Bowtie2. More than 92% of reads can be mapped back to the assembled transcriptome (Supplementary Figure 3A) suggesting the validity of the assembled transcriptome. The number of reads mapped to each transcript was obtained for differential gene expression analysis using edgeR software. Transcript abundance of the RNA-seq bioreplicates was highly correlated as revealed by Pearson’s correlation coefficient of CPM (counts per million) expression values (r > 0.98; Supplementary Figure 3B). A false discovery rate (FDR) cutoff of 0.01 was applied to select 16,488 differentially expressed genes (DEGs). Of these, 8,522 (52%) DEGs were upregulated, and 7,966 (48%) DEGs were downregulated under −N (Supplementary Table 1). GO enrichment analysis with an adjusted P-value (Padj) cutoff of 0.001 identified upregulated DEGs to be associated with the oxoacid metabolic process, serine-type endopeptidase activity, amino acid binding, and eukaryotic translation initiation factor 3 complex (Fig. 4A; Supplementary Table 2). There were multiple GO terms associated with the downregulated DEGs (Fig. 4A; Supplementary Table 2). The top three GO terms from the downregulated DEGs were thylakoids, photosynthesis, and pigment biosynthetic process (Fig. 4A; Supplementary Table 2). The GO analysis results confirmed our hypothesis that the synthesis of photosynthesis pigments was inhibited under −N.

To further analyze the DEG functions, we applied the MAPMAN tool to our data. MAPMAN analysis was performed using the Wilcoxon rank-sum test with a P-value cutoff of 0.05 (Fig. 4B; Supplementary Table 2). This analysis confirmed the GO enrichment results that photosynthesis (Bins 1, 1.1.1, and 1.1.2) was down regulated, and amino acid synthesis (Bin 13.1) was up regulated under −N (Fig. 4B; Supplementary Table 2). The MAPMAN results further revealed that the DEGs involving in nitrate metabolism (Bin 12.1) were induced by −N (Fig. 4B; Supplementary Table 2). These data signified that under −N, the cells increased their ability to take up nitrogen. Additionally, the Mapman results indicated that the glycolysis genes (Bins 4.2, 4.1.8, and 4.2.8) were up regulated, while the TAG lipase genes (Bin 11.9.2.1) and carotenoids (Bin 16.1.4) were down regulated under −N (Fig. 4B; Supplementary Table 2).

### Carbon flux shifts towards *de novo* fatty acid biosynthesis in response to −N

**Figure 5:**
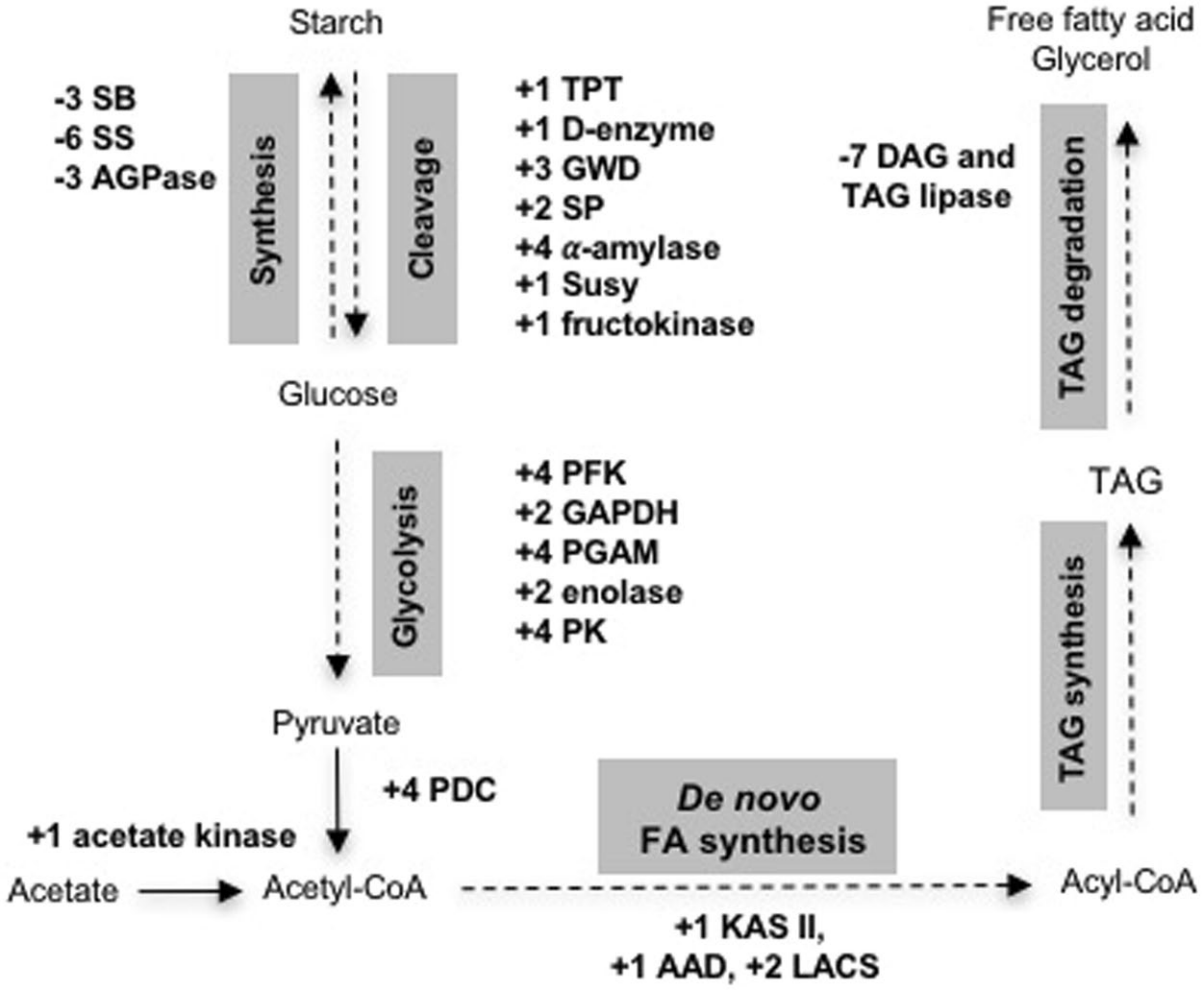
Schematic drawing of transcriptome adjustments towards TAG accumulation in *S*. *acutus*. Plus indicates transcriptional up-regulation. Minus indicates transcriptional down-regulation. Data used to generate this figure can be found in Supplementary Table 1.

We found that under −N, the expression of starch cleavage genes including *triose-phosphate transporter* (*TPT: c24129_g1_i1*), *4-α-glucanotransferase* (*D-enzyme: c740_g1_i1*), *glucan water dikinase* (*GWD: c1277_g1_i1, c10271_g33_i1,* and *c20068_g1_i1*), *starch phosphorylase* (*SP: c9904_g1_i2* and *i3*), *α-amylase* (*c412_g1_i1, c10493_g9_i2, c28729_g1_i1,* and *c33377_g1_i1*), *sucrose synthase* (*Susy*: *c8912_g1_i2*), and *fructokinase* (*c9298_g4_i1*) was induced (Fig. 5; Supplementary Table 1). In contrast, we observed the decreased expression of several genes involving in starch synthesis. These include three *ADP-glucose pyrophosphorylase* (*AGPase: c9688_g1_i1, c9688_g2_i1,* and *c24642_g1_i1*), three *starch branching enzyme* (*SB: c9616_g3_i1, c10738_g1_i1,* and *c24521_g1_i1*), and six *starch synthase* (*SS: c8813_g1_i1, c10182_g2_i1, c10540_g1_i1, c10182_g1_i1, c10182_g3_i1*, and *c15126_g1_i1*) (Fig. 5; Supplementary Table 1). In the glycolytic pathways, we found that the expression of *phosphofructokinase* (*PFK: c6362_g1_i1* and *c10466_g19_i1* and *i2*), *glyceraldehyde 3-phosphate dehydrogenase* (*GAPDH: c10641_g1_i1* and *c24212_g1_i1*), *phosphoglycerate mutase* (*PGAM: c3021_g1_i2, c9255_g2_i1, c10485_g24_i3,* and *c24459_g1_i1*), *enolase* (*c19909_g1_i1* and *c29129_g1_i1*), and *pyruvate kinase* (*PK: c4713_g2_i1, c10123_g12_i1, c10306_g22_i1*, and *c19619_g1_i1*) was induced under −N (Fig. 5; Supplementary Table 1). The up-regulation of glycolytic enzymes under −N has been documented in studies of *C. reinhardtii* and *N. oceanica*^14,15^. In agreement with the previous findings, our results suggested that under −N, *S*. *acutus* presumably activated starch degradation and glycolysis. In photosynthetic organisms, PFK functions as a key enzyme in glycolysis that catalyzes the formation of fructose-1,6-bisphosphate. GAPDH is involved in both the glycolysis and biosynthesis of glycerol which serves as a fatty acid backbone. It therefore functions as a key connector between glycolysis/gluconeogenesis and lipid metabolism^16^. Moreover, under −N, the expression of four *pyruvate dehydrogenase genes* (*PDC: c3589_g1_i1, c9239_g4_i1, c100150_g10_i1*, and *c24939_g1_i1*; Fig. 5; Supplementary Table 1), which catalyzed the conversion of pyruvate to acetyl-CoA was increased. The upregulation of PDC is correlated with an increase in lipid accumulation in higher plant^23^.

In this study, *S*. *acutus* was cultivated mixotrophically using acetate as a carbon source in combination with photosynthetic carbon fixation. In *C. reinhardtii*, the assimilation of acetate can occur as a result of the direct conversion of acetate to acetyl-CoA catalyzing by acetyl-CoA synthethase, or by a two-step process catalyzed by acetate kinases and phosphate acetyl transferase^24,25^. In *S*. *acutus*, the expression of *acetyl-CoA synthethase* (*c9609_g1_i1*, *i2*, and *i3*) was not differentially regulated under −N (Supplementary Table 1). While a *phosphate acetyl transferase* (*c24811_g1_i1*) gene was not differentially expressed, the expression of *acetate kinase* (*c29172_g1_i1*) was strongly induced under −N (Fig. 5, Supplementary Table 1), suggesting that this two-step process is possibly a main contributor to acetate assimilation in *S*. *acutus*.

Under −N, the expression of several tricarboxylic acid (TCA) genes (*aconitase* (*c3856_g2_i1*), *citrate synthase* (*c20049_g1_i1, c24189_g1_i1*, and *c28983_g1_i1*), *fumarase* (*c15399_g1_i1*), *isocitrate dehydrogenase* (*c6837_g1_i3*), *succinate dehydrogenase* (*c9370_g1_i1* and *i2*), and *succinyl-CoA ligase* (*c19990_g1_i1*)) was also up-regulated (Supplementary Table 1), implying that the acetyl-CoA derived from glycolysis could possibly be directed into the TCA cycle to produce ATP energy. Our results further demonstrated that the expression of *isocitrate lyase* (*c10384_g18_i2* and *i8*), a key enzymatic gene for the gluconeogenesis/glyoxylate cycle, was down regulated (Supplementary Table 1), indicating that gluconeogenesis might be suppressed. Two lines of evident support our findings. In diatom (*Phaeodactylum tricornutum*) cultivated under −N, the down-regulation of gluconeogenesis and glyoxylate genes is observed^26^. In *Chlorella vulgaris* cultivated under −N, the activity of isocitrate lyase strongly decreases^27^. Taken together, the reroute of carbon flux obtained from starch degradation into the precursors for de novo fatty acid synthesis could contribute to TAG accumulation in *S*. *acutus* under −N.

### Differential expression of genes encoding for enzymes in *de novo* fatty acid and TAG metabolic pathways

The first step of *de novo* fatty acid biosynthesis is the conversion of acetyl-CoA to malonyl-CoA by acetyl CoA carboxylase (ACCase). In this study, three *ACCase* genes (*c3696_g1_i1*, *c26596_g1_i1*, and *c37145_g1_i1*) were annotated from the transcriptome data; however, none were differentially expressed under −N (Supplementary Table 1). Subsequently, Malonyl-CoA-acyl carrier protein transacylase (MAT) catalyzes the conversion of malonyl-CoA to malonyl-acyl carrier protein (ACP). Our transcriptome annotated a single *MAT* gene (*c9225_g1_i1*) which was down-regulated under −N (Supplementary Table 1). The biosynthesis of C16,0-ACP (palmitoyl-ACP) from malonyl-ACP is successively catalyzed by β-ketoacyl-acyl-carrier-protein synthase (KAS) III, β-ketoacyl-ACP reductase (KAR), 3-hydroxyacyl-ACP dehydratase (HAD) and enoyl-ACP reductase (EAR)^28^. In *S*. *acutus*, two *KASIIIs* (*c10393_g1_i1* and *c33122_g1_i1*), six *KARs* (*c3547_g1_i1* and *i2*, *c3857_g1_i1*, *c3857_g2_i1*, *c17403_g1_i1*, *c21825_g1_i1*, and *c24715_g1_i1*), one *HAD* (*c20578_g1_i1*), and one *EAR* (*c11445_g1_i1*) were identified from the transcriptome data (Supplementary Table 1). In general, most enzymes in *de novo* fatty acid synthesis, including *KASIII*, *KAR*, *HAD* and *EAR*, were down-regulated or not differentially expressed under −N (Supplementary Tables 1). Once the C16,0-ACP (palmitoyl-ACP) has been formed, KASII catalyzes the elongation of the C16,0-ACP to C18,0-ACP (stearyl-ACP), which is a precursor of stearic and oleic acids. In this study, two *KASII* genes (*c7302_g1_i1* and *c10269_g28_i1*) were annotated from the transcriptome data (Supplementary Table 1). The expression of one *KASII* (*C10269_g28_i1*) was up-regulated under −N (Supplementary Table 1).

Three common products of *de novo* fatty acid synthesis are C16:0, C18:0 and C18:1^28,29^. In *Arabidopsis*, a Δ9-acyl-ACP desaturase (AAD) enzyme, namely SSI2 (encoded by *SA INSENSITIVITY OF npr1*-*5*, *AT2G43710*), catalyzes the desaturation of C18:0-ACP to C18:1-ACP^30^. In *S*. *acutus*, two *AAD* genes (*c10084_g1_i1* and *i2* and *c28773_g1_i1*) were annotated (Supplementary Table 1). We discovered that in *S*. *acutus*, the expression of one *AAD* gene (*c28773_g1_i1*) was enhanced under −N (Supplementary Table 1). This finding is consistent with the increase in C18:0 content from *S*. *acutus* TAG fraction under −N as reported by Damiani *et al*.^12^. We speculated that in *S*. *acutus*, the up-regulation of the *AAD* gene may contribute to the accumulation of oleic acid containing TAG under −N. The expression of the *fatty acyl-ACP thioesterase A* (*FAT: c10065_g3_i1*), encoding for an enzyme catalyzing the conversion of fatty acyl-ACP to free fatty acid and ACP, was down regulated in this study (Supplementary Tables 3). Once the free fatty acid was released in plastid, it can be converted to acyl-CoA by long-chain acyl-CoA synthase (LACS). Of the two annotated *LACS* genes (*c3920_g1_i1* and *c3920_g2_i1*), the expression of both was induced under −N (Supplementary Table 1), implying that the existent free fatty acids could be directed to TAG synthesis.

In *C. reinhardtii*, the expression of the key TAG synthesis genes (*glycerol-3-phosphate O-acyltransferase* (*GPAT*) and *diacylglycerol O-acyltransferase* (*DGAT*)) could be induced under −N^15,31^. In our study, we could not observe changes in the expression of *GPAT* (*c6287_g1_i1* and *c10035_g4_i1*) and *DGAT* (*c9561_g1_i1*; Supplementary Table 1). In contrast, we found that the expression of diacylglycerol (DAG) and the TAG lipase genes (*c5789_g2_i1*, *c7725_g1_i1*, *c9809_g7_i1*, *c9888_g4_i1*, *c10540_g9_i1*, *c11776_g1_i1*, and *c24853_g1_i1*) was down-regulated under −N (Fig. 5; Supplementary Table 1). Li *et al*.^32^ demonstrated that in *C. reinhardtii*, the knocking down of *CrLIP1*, a lipase that acts against diacylglycerol and polar lipids, resulted in a delay in TAG turnover. We propose that −N-induced lipid accumulation in *S*. *acutus* resulted from the inhibition of the TAG turnover by the down-regulation of DAG and TAG lipase genes.

### Validation of transcriptome data by qRT-PCR

**Figure 6:**
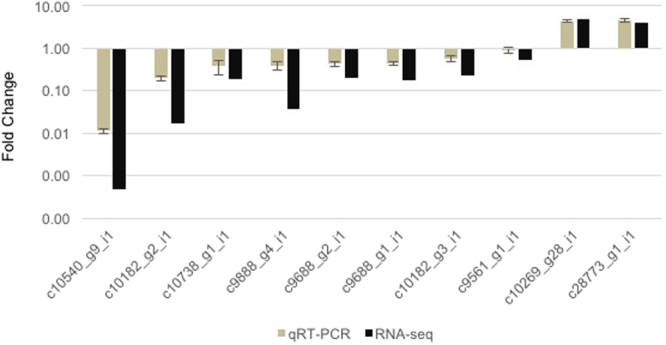
Quantitative realtime-PCR validation of the transcriptome data. Grey bars represent fold changes evaluated by quantitative realtime-PCR (data represents mean ± SE; *n* = 3). Black bars represent fold changes derived from RNA-seq data.

To verify the transcriptome results, we selected nine DEGs and one non-DEG involving starch or lipid metabolism for quantitative realtime PCR (qRT-PCR) analysis. Of the ten genes, five starch synthesis genes (*AGPases*: *c9688_g1_i1* and *c9688_g2_i1*, *SS*: *c10182_g2_i1* and *c10182_g3_i1*, and *SB*: *c10738_g1_i1*) and two *TAG lipases* (*c9888_g4_i1* and *c10540_g9_i1*) were down regulated, two lipid synthesis genes (*KASII*: *c10269_g28_i1* and *AAD*: *c28773_g1_i1*) were upregulated and *DGAT* (*c9561_g1_i1*), a key enzyme for TAG synthesis, was not differentially regulated under −N, as identified by RNA-seq and qRT-PCR (Fig. 6; Supplementary Table 1). The expression of a non-DEG, *Guanine nucleotide-binding subunit beta* (*c24199_g1_i1*), was used as a reference for the calculation of relative gene expression. qRT-PCR was performed using the same samples used for RNA-seq. These results suggest the reliability of our RNA-seq data.

### Comparative analysis of transcriptome adjustment under nitrogen starvation in oleaginous green microalgae

**Figure 7:**
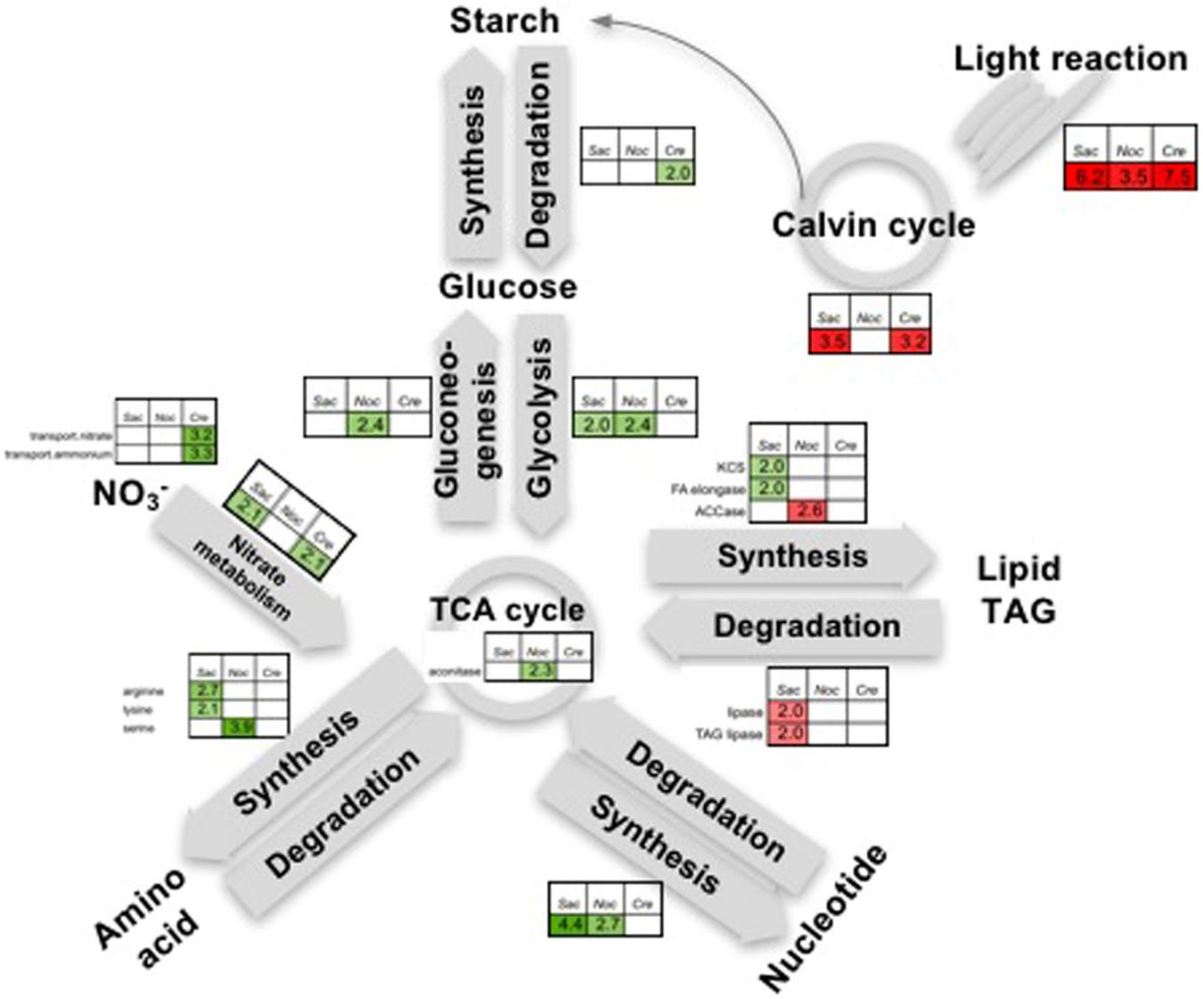
Comparative transcriptome response of oleaginous microalgae to nitrogen starvation. PAGEMAN over-representation analysis was conducted on the gene expression data (FDR < 0.01) using the Fisher method with the cutoff of <2. Green and red represents up-regulation and down-regulation, respectively. Number represent the degree of over-representation (z-score). Data used to reproduce this figure can be found in Supplementary Table 3.

To examine whether or not there is a relationship between the ability to store lipid and specific molecular mechanisms among oleaginous microalgae, we first analyzed publicly-available RNA-seq data to identify DEGs from the oleaginous models, *N. oceanica*^14^ and *C. reinhardtii*^17,33,34^, under −N (Supplementary Table 3). To obtain an overview of the comparative transcriptome analysis, we performed a PAGEMAN over-representation analysis (ORA) of DEGs using Fisher’s exact test by setting a threshold of two (Supplementary Table 3). This analysis permitted a comparison of genes with similar molecular functions. The ORA identified the down-regulation of photosynthesis genes (Bin 1) as a common response to −N (Fig. 7; Supplementary Table 3). Moreover, the up-regulation of nitrate metabolism genes was observed in *S*. *acutus* and *C. reinhardtii* (Fig. 7; Supplementary Table 3). In *C*. *reinhartdii*, the genes controlling nitrate and ammonium transport (Bins 34.4 and 34.5) were over-represented in the up-regulated DEGs (Fig. 7; Supplementary Table 3). In cells, most of the nitrogen is tied in amino acid and nucleotides. Our analysis found that the genes involved in amino acid and nucleotide metabolism were particularly over-represented in the up-regulated DEGs in *S*. *acutus* and *N. oceanica* (Fig. 7; Supplementary Table 3). We reason that the up-regulation of amino acid, nitrogen, and nucleotide metabolism genes appeared to be a general response to −N in the oleaginous microalgae.

Glycolysis genes (Bins 4.2 and 4.3) were over-represented in the case of the up-regulated DEGs of *N. oceanica* and *S*. *acutus* (Fig. 7; Supplementary Table 3) suggesting that in these oleaginous microalgae, −N resulted in an upregulation of acetyl-CoA production. Additionally, gluconeogenesis (Bin 6) and TCA (Bin 8.1.3) genes were over-represented in the up-regulated DEGs of *N. oceanica* (Fig. 7; Supplementary Table 3). The ORA analysis of the DEGs of *N. oceanica* discovered the down-regulation of ACCase (Bin 11.1.1.2), despite its ability to accumulate TAG (Fig. 7; Supplementary Table 3). Our results support the previous finding of Li *et al*.^14^ that while most of the fatty acid genes were down-regulated, genes involving in providing the carbon precursors and energy to fatty acid synthesis were up-regulated in *N. oceanica*.

The ORA analysis of the *C. reinhardtii* DEGs identified the up-regulation of photosynthesis-related carbon concentration (Bin 8.3) and major carbohydrate degradation (Bin 2.2) genes under nitrogen starvation (Supplementary Table 3), suggesting that −N could affect CO_2_ fixation and starch degradation in *C. reinhardtii*.

In *S*. *acutus*, the fatty acid elongation genes, including ketoacyl CoA synthase and fatty acid elongase (Bins 11.1.10 and 11.1.1, respectively), were specifically induced in *S*. *acutus* (Fig. 7; Supplementary Table 3). These findings implied that −N may particularly affect the hydrocarbon profile of *S*. *acutus*. To support this, in *C. reinhardtii*, a recent study demonstrated changes in hydrocarbon profile under −N cultivation^35^. Likewise, the over-representation of TAG lipase (Bin 11.9.2.1) genes from the down-regulated DEGs was exclusively found in *S*. *acutus* (Supplementary Figure 4), suggesting the differential regulation of these genes could result in lipid accumulation in *S*. *acutus*.

### Genetic engineering strategies to increase TAG accumulation in *S*. *acutus*

Our study highlights several possibilities for future investigation. The finding that a group of *DAG* and *TAG lipase* genes were down-regulated under −N, particularly in *S*. *acutus* (Fig. 7; Supplementary Figure 4; Supplementary Table 3), indicating they are potential target genes for gene knockout to increase TAG accumulation. To support this idea, previous studies have demonstrated that knocking down of *TAG lipase* genes increases the lipid content of *C. reinhardtii* and diatom (*Thalassiosira pseudonana*)^32,36^. Additional methods that have been proven to increase TAG accumulation in microalgae include enhancing fatty acid synthesis by the overexpression of *FAT* genes^37–39^ and the disruptive mutation of an *acyl-CoA oxidase* gene involving fatty acid β-oxidation^40^. Moreover, since both the synthesis of starch and TAG share carbon precursors, blocking of the starch synthesis can shift the carbon flux toward TAG accumulation. Evidently, in *C. reinhardtii*, *Dunarella tertiolecta*, and *Scenedesmus obliquus*, mutations that affected starch accumulation yielded an increase in total lipid and TAG contents following −N^21,41,42^. In our analysis, starch synthesis genes were down-regulated, while starch degradation, glycolysis and TCA genes were up-regulated, implying that changes in carbon partitioning could affect TAG synthesis under −N (Fig. 7; Supplementary Table 3). Future research should target the starch synthesis pathway to enhance carbon flux towards TAG biosynthesis in *S*. *acutus*.

## Conclusions

In this study, we demonstrated that −N was associated with increased carbohydrate and lipid accumulation in *S*. *acutus*. Under −N, the differential expression of glycolysis, starch degradation and TCA cycle genes could result in a shift of carbon flux toward fatty acid and TAG biosynthesis. Moreover, the down-regulation of TAG turnover pathway in *S*. *acutus* may be attributed to the accumulation of TAG under −N. Our study provides new insights into the molecular basis of lipid accumulation under −N in *S*. *acutus*, and opens up new revenue routes for genetic engineering to increase biodiesel production in *S*. *acutus*.

## Methods

### Growth condition, harvesting, and RNA extraction

The *Scenedesmus acutus* strain TISTR8540 was obtained from Thailand Institute of Scientific and Technological Research (TISTR; http://www.tistr.or.th/tistr_culture/). Cells were cultivated in tris-acetate-phosphate (TAP) medium under continuous light at 50 μmol photons m^−2^ s^−1^ at 25 °C with shaking. Unless otherwise indicated, log-phase cultures (3–5 × 10^6^ cells/ml) were used to inoculate fresh cultures with a starting density of 0.2 × 10^6^ cells/ml in TAP or 1 × 10^6^ cells/ml in nitrogen-free TAP (TAP-N) for all experiments. Cells were harvested by centrifugation, and pellets were kept at −80 °C for further analysis. For RNA extraction, the frozen pellets were ground in liquid nitrogen using mortar and pestle. Total RNA was extracted using Tripure Isolation Reagent (Roche) and chloroform, as described by Suttangkakul *et al*.^43^.

### Pigment analysis

Total chlorophylls and carotenoids were measured using Lichtenthaler’s method^44^. Log-phase cultures were used to inoculate in either TAP or TAP-N media at the starting density of 2 × 10^6^ cells/ml. The cell density was confirmed by counting the cells from some samples on a hemocytometer under a microscope. At appropriate time points, one-milliliter of culture was centrifuged and supernatant discarded before immediately frozen in liquid nitrogen and kept at −80 °C. For measurement, 200 mg of sea sand and 1 ml of acetone were added to the frozen pellets. The samples were then sonicated for 30 minutes using Ultrasonic Elma PNA transonic T 470/H. After centrifugation, the supernatant was collected for spectrophotometric measurement of pigments.

### Estimation of total lipids and starch

The total lipid was analyzed using the sulfo-phospho-vanillin method^45^. In brief, frozen pellets from 1.5 × 10^6^ cells were mixed with 200 μl of sulfuric acid and boiled for 10 minutes, followed by 5 minutes on ice. Five-hundred microliters of phosphovanillin were then added, and the samples were incubated at room temperature with shaking. Samples were centrifuged and the supernatant was subjected to spectrophotometric measurement at 530 nm.

For starch analysis, cultures from 20 ml of TAP and 40 ml of TAP-N media were collected at each time point. A Total Starch (AA/AMG) assay kit from Megazyme (Ireland) was used according to the manufacturer’s protocols for starch samples that also contain D-glucose.

### Nile Red staining of neutral lipid

Two hundred and fifty microliters of culture were incubated with 2.5 μl of Nile Red (Sigma) in the dark at room temperature for 30 minutes, followed by an addition of 250 μl of 8% paraformaldehyde, and another 30-minute incubation. Samples were then washed twice with 500 μl of 1% PBS by centrifugation at 8,000 rpm for 10 minutes. Ten microliters of Prolong Gold antifade reagent (Invitrogen, USA) were then added, and the samples were visualized using excitation at 488 nm and emission at 525 nm.

### High-throughput sequencing and data analysis

For each sample, 10 μg of total RNAs was used to generate a sequencing library using a Illumina^®^ TruSeq^TM^ RNA Sample Preparation Kit. Paired-end, 100 bp RNA-seq was performed on a HiSeq2500 platform. FASTQ files were generated with the base caller provided by the instrument. Quality control filtering and 3′ end trimming were analyzed using the FASTX-toolkit (http://hannonlab.cshl.edu/fastx_toolkit/index.html) and Trimmomatic software^46^, respectively. The raw read files were deposited in the NCBI SRA database under the accession numbers SRR5894887, SRR5894888, SRR5894889, and SRR5894890.

### Transcriptome analysis

The transcriptome was assembled and annotated using Trinity software^47^ and Blast2GO software^48^, respectively. The assembly was performed using a kmer value of 25 with default parameters. This Transcriptome Shotgun Assembly project has been deposited at DDBJ/EMBL/GenBank under the accession GFUP00000000. The version described in this paper is the first version, GFUP01000000. Differential gene expression analysis was performed according to Juntawong *et al*.^49^. The FASTQ files were aligned to the reference transcriptome using Bowtie2 software^50^. A binary format of sequence alignment files (BAM) was generated and used to create read count tables by the HTseq python library^51^. Differentially-expressed genes were calculated using the edgeR program^52^ with an FDR cutoff of <0.01.

Gene ontology enrichment analysis was performed in the R environment according to Juntawong *et al*.^49^. Gene annotation file was generated by the Blast2GO software. Significant GO terms were filtered by adjusted *p*-value of <0.001.

For Mapman analysis^53,54^, the mapping file was generated from the *de novo* assembled reference transcriptome using the Mercator pipeline^55^. The Mapman analysis was conducted using the Wilcoxon rank sum test with a *p*-value cut-off of <0.05.

### Comparative analysis of publicly available transcriptome data

Publicly available RNA-seq data were downloaded from the Gene Expression Omnibus (GEO) database (details listed in Supplementary Table 3). The downloaded data were mapped to the available reference genome using the Tophat2 program^56^ with a default setting. Differentially expressed genes were identified using the edgeR program using an FDR cutoff of <0.01.

For comparative transcriptome analysis, the organism-specific mapping files were generated from protein reference sequences by the Mercator pipeline^55^. Over-representation analysis was performed using the PAGEMAN program^57^ using the Fisher’s exact test with a cutoff of 2.

### Quantitative real-time PCR analysis

Genomic DNA was eliminated from the total RNA samples by treatment with DNase I (NEB, USA) according to the manufacturer’s protocol. One microgram of total RNA was used to prepare cDNA using MMuLv reverse transcriptase (Biotechrabbit, Germany) in a final volume of 20 μl. The cDNA was then diluted five times for qPCR reaction. Quantitative PCR was performed using QPCR Green Master Mix (Biotechrabbit, Germany) on a Mastercycler ep realplex 4 (Eppendorf, Germany). The PCR reaction was carried out in triplicate for each sample. Each reaction contained 1 μl of diluted cDNA, 0.5 μM of each primer, 5 μl of QPCR Green Master Mix, in a final volume of 10 μl. The PCR cycle was 95 °C for 2 min, followed by 45 cycles of 95 °C for 20 s, 60 °C for 20 s and 72 °C for 20 s. Relative gene expression was calculated using the 2^−∆∆CT^ method. The genes and primers used are shown in Supplementary Table 4.

## Electronic supplementary material

Supplementary Figures

Supplementary Table 1

Supplementary Table 2

Supplementary Table 3

Supplementary Table 4
