# Supplementary material for: De novo transcriptome analysis and gene expression profiling of an oleaginous microalga Scenedesmus acutus TISTR8540 during nitrogen deprivation-induced lipid accumulation
Source: Sci Rep. 2018 Feb 27;8:3668. doi: 10.1038/s41598-018-22080-8 (PMC5829077; doi:10.1038/s41598-018-22080-8)
Supplement: Supplementary file 1 — Supplementary Figures [file 41598_2018_22080_MOESM1_ESM.pdf]

Supplementary Figures and Legends

De novo **transcriptome analysis and gene expression profiling of an oleaginous microalga** ***Scenedesmus acutus*** **TISTR8540 during nitrogen deprivation-induced lipid accumulation**

Anchalee Sirikhachornkit, Anongpat Suttangkakul, Supachai Vuttipongchaikij, and Piyada Juntawong

Manuscript: SREP-17-48577A

Supplementary Figure 1.

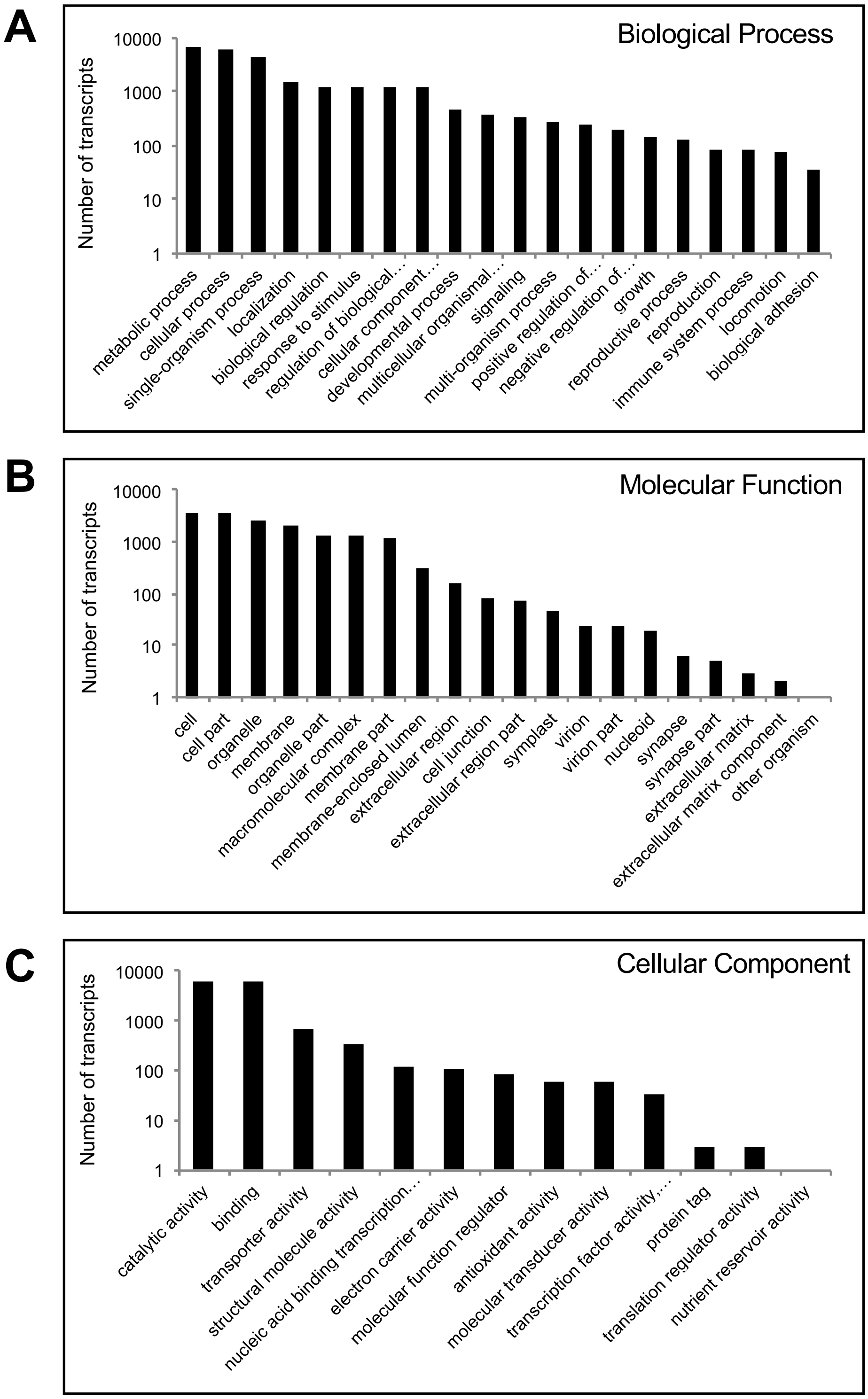

Supplementary Figure 2.

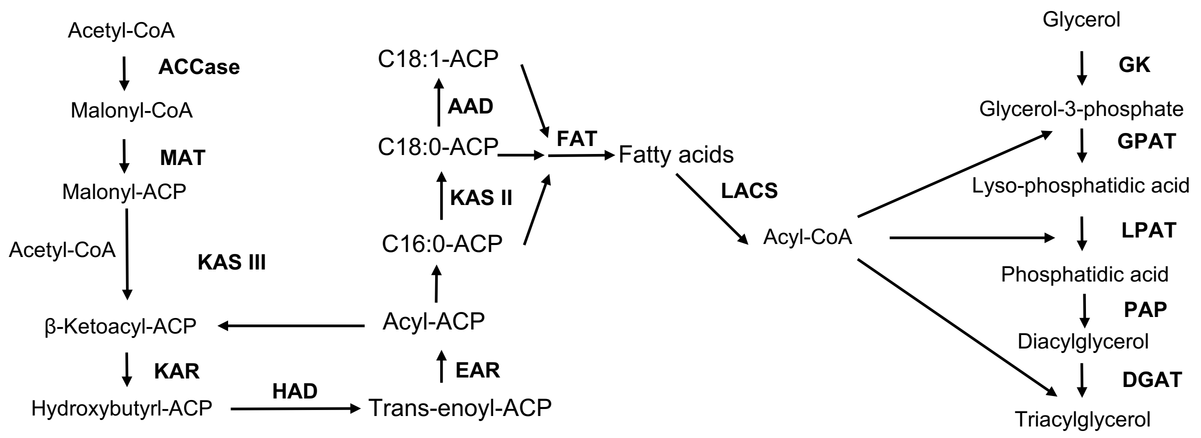

Supplementary Figure 3.

A

Sample	Read Count	Overall alignment rate
"+N rep1"	42,013,340	94.38%
"+N rep2"	45,318,070	93.71%
"-N rep1"	43,708,442	93.43%
"-N rep2"	49,650,100	92.22%

B

	"+N replicate 1"	"+N replicate 2"	"-N replicate 1"	"-N replicate 2"
"+N replicate 1"	1.000	0.988	0.535	0.505
"+N replicate 2"		1.000	0.502	0.468
"-N replicate 1"			1.000	0.989
"-N replicate 2"				1.000

Supplementary Figure 4.

	ID	logFC	Bin.ID	Bin.Name
S. acutus	c11776_g1_i1	-3.19	11.9.2.1	lipid metabolism.lipid degradation.lipases.triacylglycerol lipase
	c10540_g9_i1	-7.67	11.9.2.1	lipid metabolism.lipid degradation.lipases.triacylglycerol lipase
	c3855_g2_i1	-1.93	11.9.2.1	lipid metabolism.lipid degradation.lipases.triacylglycerol lipase
	c9888_g4_i1	-3.27	11.9.2.1	lipid metabolism.lipid degradation.lipases.triacylglycerol lipase
	c7725_g1_i1	-1.44	11.9.2.1	lipid metabolism.lipid degradation.lipases.triacylglycerol lipase
	c5789_g2_i1	-2.31	11.9.2.1	lipid metabolism.lipid degradation.lipases.triacylglycerol lipase
	c9809_g7_i1	-2.50	11.9.2.1	lipid metabolism.lipid degradation.lipases.triacylglycerol lipase
	c24853_g1_i1	-2.69	11.9.2.1	lipid metabolism.lipid degradation.lipases.triacylglycerol lipase
	c24149_g1_i1	-4.92	11.9.2.1	lipid metabolism.lipid degradation.lipases.triacylglycerol lipase
N. oceanica	CCMP1779|6228	-0.48	11.9.2	lipid metabolism.lipid degradation.lipases
	CCMP1779|7262	-0.35	11.9.2	lipid metabolism.lipid degradation.lipases
	CCMP1779|3971	0.20	11.9.2	lipid metabolism.lipid degradation.lipases
	CCMP1779|5911	0.89	11.9.2	lipid metabolism.lipid degradation.lipases
	CCMP1779|10811	-0.27	11.9.2.1	lipid metabolism.lipid degradation.lipases.triacylglycerol lipase
	CCMP1779|1271	1.64	11.9.2.1	lipid metabolism.lipid degradation.lipases.triacylglycerol lipase
C. reinhardtii	Cre01.g002400	-1.50	11.9.2	lipid metabolism.lipid degradation.lipases
	Cre03.g174900	-1.18	11.9.2	lipid metabolism.lipid degradation.lipases
	Cre07.g350000	0.85	11.9.2	lipid metabolism.lipid degradation.lipases
	Cre07.g348550	1.06	11.9.2	lipid metabolism.lipid degradation.lipases
	Cre01.g005200	1.14	11.9.2	lipid metabolism.lipid degradation.lipases
	g14829	1.15	11.9.2	lipid metabolism.lipid degradation.lipases
	g9707	1.41	11.9.2	lipid metabolism.lipid degradation.lipases
	Cre12.g504350	1.54	11.9.2	lipid metabolism.lipid degradation.lipases
	Cre01.g028250	0.88	11.9.2.2	lipid metabolism.lipid degradation.lipases.acylglycerol lipase
	Cre01.g038900	3.39	11.9.2.2	lipid metabolism.lipid degradation.lipases.acylglycerol lipase

Supplementary Figure Legends

Supplementary Figure 1. Gene ontology annotation results. Distribution of the transcripts with gene ontologies in Biological Process, Molecular Function, and Cellular Component categories.

Supplementary Figure 2. Reconstruction of *de novo* fatty acid and TAG biosynthetic pathway from transcriptome data. Bold letters indicate the enzymes used in each step. Transcript data corresponding to all enzyme groups can be found in Supplementary Table 1. Abbreviations: AAD,Δ9-acyl-ACP desaturase; ACCase, acetyl CoA carboxylase; ACP, acyl carrier protein; DGAT, diacylglycerol O-acyltransferase; EAR, enoyl-ACP reductase (NADH); FAT, fatty acyl-ACP thioesterase A; GK, glycerol kinase; GPAT, glycerol-3-phosphate O-acyltransferase; HAD; 3-hydroxyacyl-ACP dehydratase; KAR, β-ketoacyl-ACP reductase; KAS, β-ketoacyl-acyl-carrier-protein synthase; LACS, long-chain acyl-CoA synthase; LPAT, plastid 1-acylglycerol-phosphate acyltransferase; MAT, malonyl-CoA-ACP transacylase; PAP, phosphatidate phosphatase.

Supplementary Figure 3. RNA-seq mapping statistic and correlation analysis. (A) Mapping statistic. (B) Pearson's correlation analysis of CPM values from RNA-seq samples.

Supplementary Figure 4. Response of TAG lipase and other lipase genes (Bin 11.9.2 and its sub-bins) to –N. Data used to reproduce this figure can be found in Supplementary Table 3.
